# Prevalence of insomnia and related factors in individuals aged 80 years and over: a cross-sectional study

**DOI:** 10.1590/1806-9282.20251961

**Published:** 2026-06-15

**Authors:** Mehmet Beler, Yusuf Bilal Çelenk

**Affiliations:** 1 Fethi Sekin City Hospital, Department of Family Medicine – Elazığ, Turkey.; 2 Ulukent Family Health Center, Department of Family Medicine – Elazığ, Turkey.

**Keywords:** Insomnia, Aging, Aged, 80 and over, Polypharmacy, Geriatrics, Sleep disorder

## Abstract

**OBJECTIVE:**

The aim of the study was to determine the prevalence of insomnia and identify associated sociodemographic and clinical factors among individuals aged 80 years and older.

**METHODS:**

This descriptive cross-sectional study was conducted between October and November 2025 in the Advanced Age Unit of Elazığ City Hospital, Turkey. A total of 419 participants aged ≥80 years were enrolled. Sociodemographic and clinical data were collected using a structured questionnaire. The presence and severity of insomnia were assessed using the Insomnia Severity Index. An Insomnia Severity Index score ≥8 was accepted as indicative of insomnia.

**RESULTS:**

The prevalence of insomnia was 38.2%. Insomnia was significantly more frequent among women compared with men (p=0.010). Low economic status was associated with a higher rate of insomnia (p=0.033). A history of falls, hypertension, diabetes, and polypharmacy showed significant associations with insomnia (all p<0.05). Insomnia Severity Index score demonstrated a positive correlation with the number of chronic diseases (r=0.487; p<0.001) and the number of medications used (r=0.455; p<0.001).

**CONCLUSION:**

Insomnia is common among individuals aged 80 years and older and is associated with female gender, low economic status, polypharmacy, multiple chronic diseases, and a history of falls. Insomnia in older adults should not be considered a normal part of aging, but a manageable condition that affects overall well-being. Routine sleep assessment and management of underlying risk factors should be integral components of geriatric care.

## INTRODUCTION

According to World Health Organization reports, the global population aged 80 years and over is expanding rapidly, a demographic shift accompanied by an increasing burden of chronic diseases, polypharmacy, and functional decline^1^. In this context, sleep serves a critical role in preserving physical and mental well-being among older adults^2^. However, insomnia—defined as difficulties in initiating or maintaining sleep leading to impaired daytime functioning—remains one of the most prevalent and disabling disorders in this age group^3,4^. Epidemiological studies indicate that while insomnia affects 2.3–25.5% of the general adult population, its prevalence rises significantly to 13–37.8% among individuals aged over 65 years^5,6,7^.

Insomnia in older adults negatively affects sleep quality, overall health, and daily functioning. It is associated with adverse clinical outcomes such as increased mortality, cardiovascular disease, cognitive decline, depression, frailty, falls, functional loss, and greater healthcare utilization^8,9^. Therefore, recognizing and managing insomnia is essential for maintaining independence and quality of life in this population.

Individuals aged 80 years and older represent the most vulnerable segment of the elderly population due to high levels of multimorbidity, polypharmacy, and functional impairment. Despite being the fastest-growing age group, the “oldest-old” remain underrepresented in sleep research, and data on the prevalence and risk factors of insomnia in this population are limited. Addressing this gap is essential for developing targeted and clinically relevant interventions.

The aim of this study was to determine the prevalence of insomnia in individuals aged 80 years and over registered at the geriatric unit of Elazığ City Hospital and to examine its relationship with sociodemographic, clinical, and lifestyle factors.

## METHODS

This descriptive cross-sectional study was conducted in the geriatric unit of Elazığ City Hospital in eastern Turkey. The unit is the only center in the province that registers and routinely follows individuals aged ≥80 years and older, who enroll voluntarily and receive home-based medical care. Study data were collected through face-to-face patient interviews between October and November 2025.

The study population consisted of a total of 1,000 individuals registered with our unit. The inclusion criteria for the study were defined as being registered with our unit, being 80 years of age or older, and volunteering to participate in the study. Participants with a diagnosis of dementia or severe cognitive impairment documented in their medical records, endstage cancer, organ failure, or other biological, psychological, and neurological disorders that hinder reliable communication were excluded from the study.

Polypharmacy was defined as the use of five or more medications daily. Economic status was self-reported and categorized as low, medium, or high. A history of falls was defined as at least one fall within the previous 12 months.

The sample size was calculated using the formula n0=Z^2^p(1-p)/d^2^, assuming a 95% confidence level (Z=1.96), prevalence of 0.50, and a 5% margin of error. The initial result was 384 participants. After applying the finite population correction for a population of 1,000 individuals and accounting for a 10% non-response rate, the required sample size was 309. Ultimately, 419 participants were included. The study complied with the Declaration of Helsinki, written informed consent was obtained from participants or guardians, and ethical approval was granted by the Fethi Sekin City Hospital Non-Interventional Research Ethics Committee (Date: 02.10.2025; Approval No:2025/16-05).

### Insomnia Severity Index

The Insomnia Severity Index (ISI) was used to assess the severity of insomnia. Its Turkish validation was performed by Boysan and colleagues in 2011. The parameters we evaluate on the scale are: difficulty falling asleep, difficulty staying asleep, waking up too early, satisfaction with sleep patterns, impairments in daily functioning, noticeable impairments caused by sleep, and stress levels caused by sleep problems. The seven items on the scale are scored from 0 to 4. The total score obtainable from the scale ranges from 0 to 28. A score of 8 or higher is considered insomnia^10,11^.

### Statistical analysis

Data were analyzed using the Statistical Package for the Social Sciences 22.0 statistical package (IBM Corp., Armonk, NY, USA). Continuous variables were expressed as mean±standard deviation (SD) or median (minimum–maximum), and categorical variables as frequencies and percentages. The normality of data distribution was assessed using the Kolmogorov-Smirnov test. For continuous variables, the Independent Samples t-test (for normally distributed data) or Mann-Whitney U test (for non-normally distributed data) was used. Categorical variables were compared using the chi-square test. Correlations between the ISI score and continuous variables were analyzed using Spearman’s correlation analysis. A p-value of <0.05 was considered statistically significant.

## RESULTS

A total of 419 individuals aged 80 years and older were included in the study. The mean age of the participants was 85.23±3.823 years. A total of 64.2% of the participants were female (n=269) and 35.8% (n=150) were male. The sociodemographic characteristics of the participants are presented in Table 1.

**Table 1:** Sociodemographic characteristics of geriatric individuals.

	Number	%
Age		
80–84	195	46.5
85–90	169	40.3
90 and above	55	13.1
Gender		
Female	269	64.2
Male	150	35.8
Education level		
Illiterate	224	53.5
Primary education	155	37.0
Middle school/high school	36	8.5
College/university	4	1.0
Family and social life		
Lives alone	64	15.3
Living with spouse	129	30.8
Lives with his spouse and children	226	53.9
Economic status		
Poor	53	12.6
Average	329	78.5
Good	27	8.8
Type of residence		
Detached house	63	15.0
Apartment	356	85.80
BMI		
Underweight	65	15.5
Normal	213	50.8
Pre-obese	132	31.5
Obese	9	2.1
History of falls		
None	318	75.9
Yes	101	24.1
Hearing problem		
No	223	55.6
Yes	186	44.4
Incontinence		
No	227	54.2
Yes	192	45.8

BMI: body mass index.

The prevalence of insomnia in the study population was 38.2% (n=160). The mean ISI score was 10.74±6.40. Themedian number of medications used by participants was 5.00 (0.00–12.00), and the median number of current chronic diseases was 2.00 (0.00–8.00). Clinical characteristics are provided in Table 2.

**Table 2:** Clinical characteristics of participants.

	Median number	Min–max percentage
Number of medications used	5.00	(0.00–12.00)
Number of chronic diseases	2.00	(0.00–8.00)
Polypharmacy status		
None	200	47.7
Yes	219	52.3
Hypertension		
No	152	36.3
Yes	267	63.7
Diabetes		
No	327	78.0
Yes	92	22.0
CHD, CAD, arrhythmia, and dyslipidemia		
No	83	19.8
Yes	336	80.2
COPD		
No	390	93.1
Yes	29	6.9
Asthma		
No	381	90.9
Yes	38	9.1
Insomnia		
None	259	61.8
Yes	160	38.2
Insomnia Severity Scale	10.74±6.400	(1.00–28.00)

CHD: coronary heart disease; CAD: coronary artery disease; COPD: chronic obstructive pulmonary disease.

In our study, the prevalence of insomnia was significantly higher in the 80–84 age group (55.0%) compared to other age groups (p=0.021). The rate of insomnia was significantly higher in women than in men (71.9 and 28.1%, respectively; p=0.010). The rate of insomnia was higher among those with poor economic status (p=0.033). Insomnia was significantly higher among individuals with a history of falls (p=0.027). The presence of hypertension (p<0.001) and diabetes (p<0.001) was found to be statistically significantly associated with insomnia; no significant association was found with hearing problems, incontinence, or Congestive Heart Failure. Polypharmacy was significantly more common in the insomnia group (p<0.001) (Table 3).

**Table 3:** Comparison of participants’ sociodemographic and biological characteristics with insomnia.

Özellikler	No Insomnia	Insomnia	p
Age			
80–84	107 (41.3)	88 (55.0)	**0.021**
85–89	113 (43.6)	56 (35.0)	
90 and above	39 (15.1)	16 (10.0)	
Gender			
Female	154 (59.5)	115 (71.9)	**0.010**
Male	105 (40.5)	45 (28.1)	
Economic status			
Poor	26 (10.0)	27 (16.9)	**0.033**
Moderate	214 (82.6)	115 (71.9)	
Good	19 (7.3)	18 (11.3)	
Fall history			
No	206 (79.5)	112 (70.0)	**0.027**
Yes	53 (20.5)	48 (30.0)	
Incontinence			
No	117 (45.2)	64 (40.0)	**0.299**
Yes	142 (54.8)	96 (60.0)	
Polypharmacy			
None	154 (59.5)	46 (28.7)	**<0.001**
Yes	105 (40.5)	114 (71.3)	
BMI			
Underweight	46 (17.8)	19 (11.9)	**0.229**
Normal	134 (51.7)	79 (49.4)	
Pre-obese	74 (28.6)	58 (36.3)	
Obese	5 (1.9)	4 (2.5)	
CHD, CAD, arrhythmia, and dyslipidemia			
None	49 (18.9)	34 (21.3)	**0.561**
Yes	210 (81.1)	126 (78.8)	
Hypertension			
No	111 (42.9)	41 (25.6)	**<0.001**
Yes	148 (57.1)	119 (74.4)	
Diabetes mellitus			
No	222 (85.7)	105 (65.6)	**<0.001**
Yes	37 (14.3)	55 (34.4)	

CHD: coronary heart disease, CAD: coronary artery disease; BMI: body mass index. The bold values indicate statistical significance (p<0.05).

No statistically significant relationship was found between the ISI score and age and body mass index (BMI) (r=-0.082; p=0.095 and r=0.055; p=0.265, respectively). In contrast, a positive and statistically significant correlation was found between the number of chronic diseases (r=0.487; p<0.001), the number of medications used (r=0.455; p<0.001), and the ISI score.

## DISCUSSION

This study specifically focused on individuals aged 80 years and older, a subgroup that is often underrepresented in sleep research despite its clinical vulnerability. We found that insomnia was highly prevalent in this age group, affecting 38.2% of participants, a rate higher than that reported in many studies involving younger elderly populations. This difference may be explained by the inclusion of mild insomnia symptoms assessed using the ISI and the higher burden of comorbidity and frailty among individuals registered in a home healthcare unit. Given the limited data available for the “oldest-old,” our findings contribute to a clearer understanding of the burden and characteristics of insomnia in this rapidly growing population.

Insomnia was most common in the 80–84 age group and decreased in those aged ≥90. This may indicate a “survivor effect,” suggesting that individuals who reach advanced age may be more resilient or have better coping mechanisms for sleep disturbances. Alternatively, underreporting due to cognitive decline or perceiving poor sleep as part of normal aging may explain this pattern. Because age influences sleep in a multifaceted way, further studies are needed to clarify sleep characteristics in the oldest-old.

When evaluated from a sociodemographic perspective, our study found that factors such as female gender and low socioeconomic status were associated with insomnia. Women constituted approximately two-thirds of the group diagnosed with insomnia, and the prevalence of insomnia in women was significantly higher than in men (p=0.010). Current studies also show that the prevalence of insomnia in the elderly population is higher in women than in men. Factors such as postmenopausal hormonal changes, depression, anxiety, chronic pain, and socioeconomic disadvantages may contribute to the high risk of insomnia in women^9,12,13^. Socioeconomic variables also affected the prevalence of insomnia. The prevalence of insomnia was found to be higher among participants with poor economic status. This is consistent with studies showing that socioeconomic disadvantage is associated with poorer sleep outcomes in older age. Limited health literacy, difficulties accessing healthcare services, and lifelong stress burden may contribute to this relationship^14,15^.

Insomnia was strongly associated with polypharmacy and chronic disease burden. More than 71% of participants with insomnia used ≥5 medications compared with 41% of those without insomnia (p<0.001). Polypharmacy is reported in 32% of older adults and may contribute to sleep disturbances through drug interactions and adverse effects^16^. Insomnia was also more common in individuals with hypertension and diabetes (both p<0.001), consistent with studies showing higher insomnia rates in chronic metabolic and cardiovascular diseases^17,18^. No significant association was found with Chronic Obstructive Pulmonary Disease (COPD), asthma, heart failure, or incontinence, possibly due to smaller subgroup sizes or symptom underreporting.

In our study, the prevalence of insomnia was significantly higher in older adults with a history of falls (p=0.027). This finding is consistent with existing evidence that sleep disorders increase the risk of falls in older adults^19,20^. On the other hand, sleep quality may also deteriorate in frail individuals with a history of falls due to pain or limited mobility; therefore, the relationship may be bidirectional. This finding suggests that insomnia and falls should be evaluated together. Improving sleep quality or reducing sedative medications may reduce the risk of falls; however, this assumption needs to be confirmed by prospective studies^21^.

Given the adverse consequences of insomnia in older adults, the clinical significance of our findings is further highlighted. Insomnia is closely associated with depression, cognitive decline, reduced quality of life, and increased mortality^12^. In a cohort of over 36,000 elderly veterans, those diagnosed with insomnia were shown to have a higher risk of all-cause mortality during approximately six years of follow-up^8^.

### Clinical implications

The findings of this study highlight the clinical relevance of insomnia in individuals aged 80 years and older, particularly in geriatric and primary care settings. Insomnia was found to be common in this age group and closely associated with polypharmacy, multimorbidity, female gender, low socioeconomic status, and a history of falls, defining a recognizable high-risk profile for targeted assessment. In routine practice, sleep problems in the oldest-old are often overlooked or considered a normal part of aging; however, our results suggest that brief screening with practical tools such as the ISI may be especially useful in patients with multiple chronic conditions or those using five or more medications. Early identification of insomnia may facilitate medication review, avoidance of inappropriate sedative use, and implementation of non-pharmacological interventions, while the association with falls underscores the potential contribution of sleep assessment to fall prevention, functional safety, and overall quality of life.

### Limitations

This study has several limitations. First, its cross-sectional design prevents the establishment of causal relationships between insomnia and the identified risk factors. Second, the study was conducted in a single center with individuals voluntarily registered in a home healthcare unit, which may limit the generalizability of the findings to the broader elderly population (selection bias). Third, data regarding falls, sleep patterns, and economic status were based on self-reports, which introduces the possibility of recall bias. Finally, specific mental health scales for depression and anxiety, which are known to strongly influence sleep, were not employed; however, patients with communication-hindering psychiatric disorders were excluded.

## CONCLUSION

This study highlights that insomnia is a highly prevalent condition among individuals aged 80 years and over, affecting nearly four out of ten individuals. It is closely linked to modifiable and manageable factors such as polypharmacy, chronic disease burden, and economic status, as well as critical outcomes like falls. These results suggest that management of insomnia in the oldest-old requires a holistic, multidisciplinary approach that goes beyond symptom control. Future health policies and clinical guidelines should prioritize routine sleep screening and integrated care strategies specifically tailored for this rapidly growing demographic to improve their quality of life and safety.

## Data Availability

The datasets generated and/or analyzed during the current study are available from the corresponding author upon reasonable request.
